# Induced Sputum Assessment in New York City Firefighters Exposed to World Trade Center Dust

**DOI:** 10.1289/ehp.7233

**Published:** 2004-09-22

**Authors:** Elizabeth M. Fireman, Yehuda Lerman, Eliezer Ganor, Joel Greif, Sharon Fireman-Shoresh, Paul J. Lioy, Gisela I. Banauch, Michael Weiden, Kerry J. Kelly, David J. Prezant

**Affiliations:** ^1^Institute for Pulmonary and Allergic Diseases, and; ^2^National Laboratory Service for Interstitial Lung Diseases, Tel-Aviv Sourasky Medical Center, Tel-Aviv, Israel; ^3^Sackler Faculty of Medicine, Tel Aviv University, Tel-Aviv, Israel; ^4^National Institute of Occupational and Environmental Health, Raanana, Israel; ^5^Department of Geophysics and Planetary Sciences, Tel Aviv University, Tel-Aviv, Israel; ^6^Institute of Chemistry, Hebrew University of Jerusalem, Jerusalem, Israel; ^7^Environmental and Occupational Health Sciences Institute of New Jersey, New Brunswick, New Jersey, USA; ^8^NYC Fire Department Bureau of Health Services, New York, New York, USA; ^9^Montefiore Medical Center, Albert Einstein College of Medicine, Bronx, New York, USA; ^10^Pulmonary Division, New York University School of Medicine, New York, New York, USA

**Keywords:** firefighters, inflammation, inhalation exposure, particulates, sputum, World Trade Center

## Abstract

New York City Firefighters (FDNY-FFs) were exposed to particulate matter and combustion/pyrolysis products during and after the World Trade Center (WTC) collapse. Ten months after the collapse, induced sputum (IS) samples were obtained from 39 highly exposed FDNY-FFs (caught in the dust cloud during the collapse on 11 September 2001) and compared to controls to determine whether a unique pattern of inflammation and particulate matter deposition, compatible with WTC dust, was present. Control subjects were 12 Tel-Aviv, Israel, firefighters (TA-FFs) and 8 Israeli healthcare workers who were not exposed to WTC dust. All controls volunteered for this study, had never smoked, and did not have respiratory illness. IS was processed by conventional methods. Retrieved cells were differentially counted, and metalloproteinase-9 (MMP-9), particle size distribution (PSD), and mineral composition were measured. Differential cell counts of FDNY-FF IS differed from those of health care worker controls (*p* < 0.05) but not from those of TA-FFs. Percentages of neutrophils and eosinophils increased with greater intensity of WTC exposure (< 10 workdays or ≥ 10 workdays; neutrophils *p* = 0.046; eosinophils *p* = 0.038). MMP-9 levels positively correlated to neutrophil counts (*p* = 0.002; *r* = 0.449). Particles were larger and more irregularly shaped in FDNY-FFs (1–50 μm; zinc, mercury, gold, tin, silver) than in TA-FFs (1–10 μm; silica, clays). PSD was similar to that of WTC dust samples. In conclusion, IS from highly exposed FDNY-FFs demonstrated inflammation, PSD, and particle composition that was different from nonexposed controls and consistent with WTC dust exposure.

In the aftermath of September 11th, the clouds of dust and smoke that stood for days in place of the World Trade Center’s (WTC) twin towers raised serious health concerns among exposed workers and residents. The Fire Department of New York City (FDNY) operated a continuous rescue/recovery effort from 11 September 2001 through May 2002. Nearly every FDNY firefighter (FDNY-FF) worked at the site during the first weeks, reporting numerous exposures to airborne particulates and products of combustion/pyrolysis [Centers for Disease Control and Prevention (CDC) 2002a] that have since been implicated in the development of “WTC cough,” airways obstruction, and inflammatory bronchial hyperreactivity (Banauch et al. 2003; Feldman et al. 2004; Prezant et al. 2002). Appropriate respiratory protection was not readily available in the first week (CDC 2002b). Firefighters were not the only ones affected. Respiratory symptoms and pulmonary dysfunction has been reported in other WTC rescue workers (Safirstein et al. 2003; Saltzman et al. 2004; Skloot et al. 2004) and in Manhattan residents living near the site. (CDC 2002c; Szema et al. 2004).

Environmental site studies after the collapse reported concentrations of airborne and respirable particulates ranging up to 100 mg/m^3^ and 1 mg/m^3^, respectively (CDC 2002a). Analysis of settled WTC dust samples collected 5 and 6 days postcollapse from areas east of the WTC revealed a complex mixture of particulate matter and combustion/pyrolysis products, composed mostly of building debris fibers (e.g., mineral wool, fiberglass, asbestos, wood, paper, cotton) contaminated with polycyclic hydrocarbons (Landrigan et al. 2004; Lioy et al. 2002). More than 90% of the particles in these bulk samples were > 10 μm in diameter and many were fibers with widths < 5 μm and lengths > 10 μm. Further, many were caustic cement particles with a pH of 9–11 (Landrigan et al. 2004; Lioy et al. 2002).

Bronchoalveolar lavage (BAL) recovered significant quantities of fly ash, degraded fibrous glass, and asbestos fibers along with evidence for a significant inflammatory response (70% eosinophils and increased levels of interleukin-5) in one FDNY-FF hospitalized with acute eosinophilic pneumonitis several weeks after WTC exposure (Rom et al. 2002). Although BAL is an important diagnostic tool (Davison et al. 1983; Dodson et al. 1991; Rom et al. 2002), it is an invasive procedure unsuitable for screening or repeated follow-up evaluations after exposure to dusts or combustion/pyrolysis products. In fact, no FDNY-FF has agreed to enroll in a BAL screening program.

Induced sputum (IS) provides a non-invasive alternative method to study respired particulate matter and the lung’s inflammatory response (Fireman et al. 1999a; Maestrelli et al. 1994; Marek et al. 2001; Quirce et al. 2001). Qualitative and quantitative analysis of chemical particles among silica and hard metal workers showed similar patterns when recovered by IS and BAL (Fireman et al. 1999a). IS analyzed by scanning electron microscope (SEM) has demonstrated dust exposures in patients with occupational lung diseases (Cohen et al. 1999; Fireman et al. 2004a; Lerman et al. 2003b; Paris et al. 2002), and IS analyzed by particle size distribution (PSD) has shown significant differences between workers with and without exposure to hazardous dust (Lerman et al. 2003b).

This study is the first to use IS methodology to assess respired particulate matter and the inflammatory response of the lung after exposure to WTC dust. Our objective was to determine if IS collected from highly exposed FDNY-FFs 10 months after the collapse demonstrates a unique pattern of inflammation and particulate matter deposition compatible with WTC dust. Inflammation was assessed by differential cell counts and by measuring metalloproteinase-9 (MMP-9), a cytokine involved in airways inflammation (Montano et al. 2004) and remodeling (Atkinson and Senior 2003). Particulate matter was assessed by size distribution, mineral composition, and by comparison to settled dust samples collected at the WTC site.

## Methods

### Study population.

Ten months after the WTC collapse, we studied 39 male FDNY-FFs who worked at the WTC, 12 male firefighters who lived in Tel-Aviv, Israel (TA-FF), and 8 male Israeli hospital workers, all apparently free of respiratory disease. Firefighters were recruited from those undergoing medical monitoring during June 2002. The only inclusion criterion was that firefighters must have worked in the WTC dust cloud the morning of 11 September 2001. Current or past tobacco smokers were excluded. IS induction was voluntary, and all were informed about the ongoing research. For FDNY-FFs, cumulative WTC exposure was measured in workdays reported on a self-administered questionnaire, followed by a confirmatory interview before IS induction. The Institutional Review Boards at Tel-Aviv Medical Center and Montefiore Medical Center approved this study.

### Sputum induction and processing.

IS was obtained at the FDNY Bureau of Health Services as previously described (Fireman et al. 1999a, 1999b; Lerman et al. 2003b). After pretreatment with a short acting beta-2 agonist, 3% saline was administered by nebulizer (U1 Ultrasonic Nebulizer; Omron HealthCare, Henfield, West Sussex, UK) for up to 20 min while subjects were encouraged to cough and expectorate sputum into a sterile container. Samples were stored at 4°C and processed within 3 hr. All portions with little or no squamous epithelial cells (rich nonsquamous epithelial cell fraction considered to originate from the lower respiratory tract, hereafter referred to as “plugs”) were collected using the selection plug method and processed as previously described (Fireman et al. 1999b; Pin et al. 1992; Popov et al. 1994). Briefly, plugs were selected and treated with dithiothreitol [DTT (Sputalysin); Calbiochem Corp., San Diego, CA, USA]. The cell suspension was filtered through a 52-μm nylon gauze (BNSH Thompson, Scarborough, Ontario, Canada) and the effect of DTT was stopped by diluting the suspension with phosphate-buffered solution to a volume equal to the sputum plus DTT. After centrifugation, the supernatants were deep frozen for consecutive measurement of MMP-9. The pellets were resuspended and cytospinned (Shandon Southern Instruments, Sewickley, PA, USA), and the slides were stained with Giemsa. We counted 200 nonsquamous cells; the results were expressed as a percentage of the total nonsquamous count. Each slide was read by two independent readers.

### Differential cell counts.

Samples from the cellular fraction were resuspended and processed to cytospin slides, centrifuged, air-dried, and stained with Giemsa. Differential cell counts were measured by scanning cytospin slides with high power (×500) magnification. We counted 200 nonsquamous cells and expressed cell differentials as percentages of total nonsquamous cell counts.

### Measurement of MMP-9 ELISA.

We determined the absolute value of MMP-9 in IS supernatants using an ELISA commercial kit (R&D Systems Inc., Minneapolis, MN, USA). MMP-9 values expressed total levels of active and pro-MMP-9 (0.1–25 ng/mL). We performed spike experiments using 10 and 20 ng/mL pure MMP-9 protein to assess the efficacy of recovery. We found that only 10–12% of protein was equally denatured in the pure protein and in all IS samples by DTT. No other metabolites were measured in the study.

### Particle examination.

After separation of the plugs and viscous materials, all fractions of IS were preserved in 10% formalin and stored at 4°C until analysis of mineral particles. We used the samples containing both extracellular and intracellular particles for the SEM analysis; samples were treated with 14% formamide solution and filtered onto a 0.8-μm carbon-coated Nuclepore filter (Millipore Filter Corp., Bedford, MA, USA). Particles with a diameter > 0.4 μm were analyzed by a JEOL 840 SEM (JEOL Ltd., Hertfordshire, UK) equipped with a Link 10,000 energy-dispersive system (EDS; Link Oxford Analytical Instruments, Oxford, UK). The spectrometer of the EDS system separated the elements according to energy rather than wavelength. In addition, we used a petrographic microscope to identify minerals (Fireman et al. 1999a, 1999b). We assessed the size and shape of the particles from the rich cell fraction of the processed plugs with a Cis-100 Analyzer and the analyzer’s video channel (Ankersmid, Yokneam, Israel) (Fireman et al. 1999b) using a PSD method in the range of 0.5–3,600 based on the time of transition (Aharonson et al. 1986; Cohen et al. 1999; Pin et al. 1992) theory where the duration of interaction between beam and particle provides a direct measurement of each particle’s size. A heliumneon laser beam interfered with the intracellular particles, and the signal was recorded. We performed dynamic shape characterization using image analysis techniques. Measurements were performed on 2 drops of a suspension of sputum cells (10^6^ cells/mL) introduced into a quartz cuvette containing stirred water for IS samples and water with glycerol (1:1) for the WTC settled dust sample collected 7 days postcollapse on Cortlandt Street, one block east of the WTC. Each result was an average of three consecutive measurements.

### Statistical analysis.

Demographic comparisons between groups were performed by parametric or nonparametric (Kruskal-Wallis) analysis of variance (ANOVA). FDNY-FFs were analyzed according to cumulative exposure days using a continuous scale and dichotomized based on the median (< 10 days vs. 10 days). Groups were compared by independent *t*-test, Mann-Whitney test, and chi-square test. In addition, group means of all percent cells and particle size, adjusted for MMP9, were compared by a one-way analysis of covariance with the natural logarithm of MMP-9 as covariate. Natural logarithm transformation was applied to MMP-9 because of its skewed distribution (Ln MMP-9). The association between percent parameters was evaluated by Pearson correlation coefficients. For all tests, *p*-values < 0.05 were considered statistically significant. The data were analyzed using Statistical Package for the Social Sciences (SPSS) for Windows software, Version 11.0 (SPSS, Chicago, IL, USA).

## Results

### Demographic characteristics.

We found no significant differences in age between the FDNY-FFs and the TA-FFs (37.4 vs. 36.6 years; *p* = 0.109, respectively) or their respective fire-fighting work tenure (17.5 vs. 13.5 years; *p* = 0.399, respectively). The average age of the nonfirefighter controls was 41.6 years. All 39 FDNY-FFs were caught in the WTC dust cloud during the morning of 11 September 2001, representing the highest acute exposure group as defined by arrival time (Banauch et al. 2003; Feldman et al. 2004). The period of cumulative WTC work exposure for the FDNY-FF group varied from 1 to 75 days (mean of 20.2 days), with all but 2 of the FDNY-FFs working at the WTC for ≥ 2 days. We found no differences in age or FDNY work years when we separated the group based on the median of cumulative WTC work-site exposure time: < 10 days (*n* = 23) or 10 days (*n* = 16).

Differential cell counts and MMP-9 levels in sputum samples. We performed IS differential cell counts in 36 of the 39 FDNY-FFs, 12 of 12 TA-FFs, and 8 of 8 controls. The firefighter groups were significantly different from the nonfirefighter controls, but we found no significant differences between the firefighter groups (Table 1). Differential counts for neutrophils and eosinophils increased with cumulative WTC workday exposure intensity (dichotomized to < 10 or 10 WTC workdays) (Table 2).

**Figure 1:**
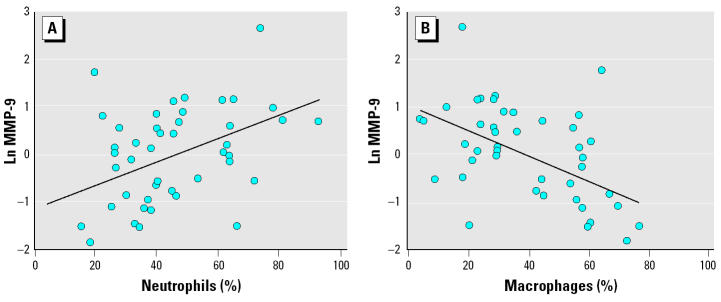
Correlation between concentrations of Ln MMP-9 and the percentage of neutrophils (*A*) and the percentage of macrophages (*B*) in induced sputum from all samples (*n* = 56). Values are expressed as the percentage of 200 cells as described in “Methods.”

We measured MMP-9 levels in IS supernatants retrieved from sample preparations of 25 of 39 FDNY-FFs, 12 of 12 TA-FFs, and 8 of 8 controls. There was a trend for higher levels in the FDNY-FFs vs. the TA-FFs (2.23 ng/mL versus 1.21 ng/mL; *p* = 0.057), and the combined levels for both groups were significantly higher than for the controls (0.30 ng/mL; *p* = 0.0001). Independent of exposure group, the levels of MMP-9 were positively correlated to the percentage of neutrophils (*r* = 0.449, *p* = 0.002; Figure 1A) and negatively correlated to the percentage of macrophages (*r* = 0.488, *p* = 0.001; Figure 1B).

### Particle size distribution.

**Figure 2:**
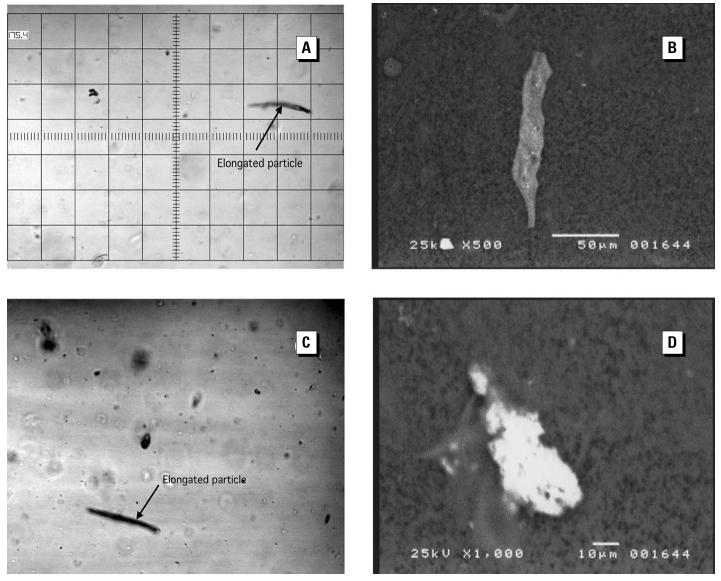
Size and shape of four different particles found in an FDNY-FF specimen. Images (*A* and *C*) and shape of particles analyzed by a Cis-100 analyzer. Images (*B* and *D*) and shape of particles analyzed by X-ray spectrum. See “Methods” for details.

We determined PSD in 35 of 39 FDNY-FF samples (4 samples were eliminated because of contamination before measurement) and 12 of 12 TA-FF samples. Table 3 shows that more of the FDNY-FF samples contained a higher percentage of particles > 2 μm (*p* = 0.0001) and > 5 μm (*p* = 0.0001) compared with the TA-FF samples. We found no significant differences in PSD measurements when they were correlated to cumulative WTC exposure (tested by a continuous or a dichotomized 10-day analysis). Most of the particles showed an irregular shape (Figure 2B,D).

**Figure 3:**
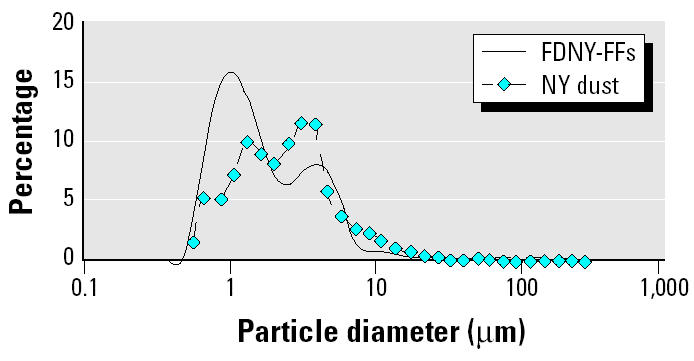
Comparison of the size distribution of particles between IS samples (*n* = 39) from FDNY-FFs and the settled material collected on Cortlandt Street, one block east of the WTC 7 days after the WTC collapse. Measurements were performed as described in “Methods.”

We also demonstrated compatibility between the PSD of the settled raw dust samples from Cortlandt Street (located one block east of the WTC) collected 7 days after the collapse with measurements from the IS of FDNY-FFs (*n* = 39; Figure 3). Both PSD curves showed similar patterns for particles > 4.37 μm in diameter, but the curve for the FDNY-FF IS showed a left shift compared to the settled raw dust material, indicating a higher proportion of small particles in the lung than in bulk samples collected from settled dust. This would be consistent with the fact that the largest dust particles do not efficiently penetrate past the nasopharyngeal region. An inversion between both curves can be observed where they intersect at 7.19 μm.

### Chemical and mineralogic analysis of particles.

**Figure 4:**
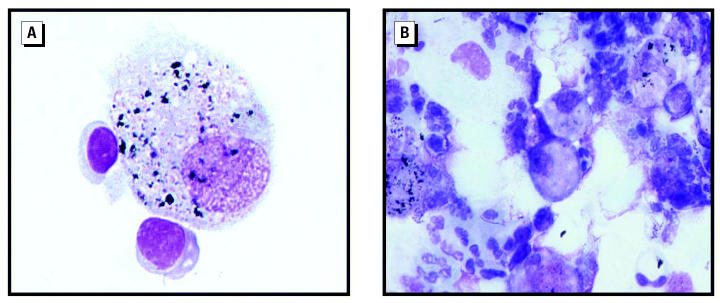
Intracellular phagocytized particles in a Giemsa-stained cytospin preparation from the IS sample of an FDNY-FF exposed to WTC dust. (*A*) Image showing a single macrophage with intracellular particles and two adjacent lymphocytes. (*B*) Image showing a mixed cell population with macrophages and intracellular particles. Light microscopy; magnification, ×100.

**Figure 5:**
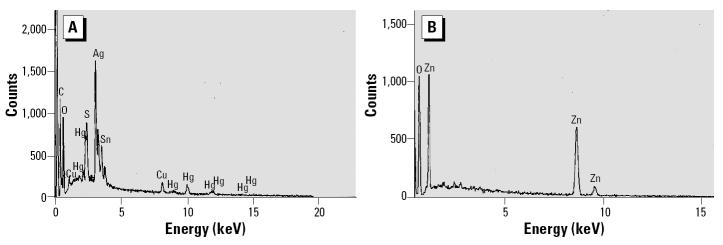
X-ray spectra of representative particles identified as Cu and Hg and Ag (*A*) and Zn (*B*) in FDNY-FF sample 35.

Chemical and mineralogic analyses were performed on IS samples from 4 FDNY-FFs and 2 TA-FFs randomly chosen from high quality specimens. Chemical analysis of the FDNY-FF samples revealed many elements, for example, titanium, zinc, mercury, gold, tin, and nickel (Table 4), which were present as metal alloys or metal oxides in abundant large particle sizes (range 1–50 μm). The shape varied from irregular (multiple angles) to spherical. In the TA-FF samples, a few smaller particles (1–10 μm) of silica and clays were found that are more typical of normal Tel-Aviv soil than of pollution. Intracellular particles are shown in Figure 4. Figure 5 shows X-ray spectrums of representative particles identifying them as zinc, copper, silver, and mercury.

## Discussion

In FDNY-FFs—all highly exposed to WTC dust and combustion/pyrolysis products during the morning of the collapse and nearly all with additional cumulative workday exposures to WTC dust—IS analysis were significantly different from controls, in inflammation (percentages of neutrophils and eosinophils that increased with exposure intensity; increased MMP-9) and particulate matter deposition (a shift in PSD toward larger size particles and chemical/mineral analysis consistent with WTC dust).

It is not surprising to find abnormalities in lung particulate matter and inflammation months after exposure. After chronic occupational exposures, IS has shown increased eosinophil counts in sensitized asthmatics (Maestrelli et al. 1994; Quirce et al. 2001) and in asbestos (Paris et al. 2002), radon (Marek et al. 2001), and uranium (Marek et al. 2001) workers. Asbestos bodies (Paris et al. 2002) and particles with abnormal chemical compositions have been observed in IS years after exposure (Cohen et al. 1999; Fireman et al 1999a, 1999b; Lerman et al. 2003a).

Firefighters are exposed to numerous irritants (e.g., combustion and pyrolysis products, particulate matter), and such exposures have the potential to alter lung permeability (Bergstrom et al. 1997; Burgess et al. 2001). Inflammatory changes have been documented in BAL from nonsmoking firefighters compared with healthy volunteers (Bergstrom et al. 1997; Burgess et al. 2001). In a previous case report (Rom et al. 2002), we found an eosinophilic inflammatory response (70% of total cell count) and increased levels of interleukin-5 in the BAL of an FDNY-FF with acute pneumonitis several weeks after repeated WTC dust exposures. The present study is the first to use IS to characterize differential cell counts, inflammation, particle size deposition, and composition in subjects with WTC exposures. It is also the first to report these findings in firefighters. IS from FDNY-FFs and TA-FFs, all never smokers, had increased percentages of eosinophils and neutrophils compared with healthy nonfirefighter controls. For FDNY-FFs, a significant dose–response relationship was demonstrated, with the proportions of neutrophils and eosinophils increasing as cumulative WTC exposure intensity increased (measured in number of days working at WTC). Neutrophils and eosinophils are important components of the inflammatory cascade responsible for airway inflammation, injury, and remodeling (Azadniv et al. 2001; Lemiere et al. 2001; Woodruff et al. 2001).

The matrix metalloproteinases (MMPs) are a family of zinc- and calcium-dependent endopeptidases with a central role in inflammation and combined ability to degrade components of connective tissue matrices (Murphy and Docherty 1992). MMPs are synthesized and secreted by connective tissue and some hematopoietic cells, and are known to be important mediators of airway inflammation, remodeling, and pulmonary injury (Cataldo et al. 2002; Li et al. 2002). We chose to measure MMP-9 because it *a*) plays an important role in neutrophil recruitment to the lung (Li et al. 2002); *b*) is detectable in IS with reliable and reproducible results (Cataldo et al. 2002; Fireman et al. 2004a); and *c*) is increased in IS from workers exposed to hazardous dust (Fireman et al. 2004b). We found a trend for higher levels of MMP-9 in IS samples from FDNY-FFs than in those from TA-FFs, and both groups were significantly higher than nonfirefighter controls. Moreover, we found a positive correlation between accumulation of neutrophils and MMP-9 levels. In these non-smoking firefighters, the increase in MMP-9 levels provides biochemical evidence for exposure-related immune activation in the lung, complementing the evidence from IS differential cell counts. Persistent inflammation, 10 months after the WTC collapse, is consistent with the clinical findings of new and persistent cough, airway hyperreactivity, and asthma previously reported in FDNY-FFs (Banauch et al. 2003; Feldman et al. 2004; Prezant et al. 2002) and other rescue workers (Safirstein et al. 2003; Saltzman et al. 2004; Skloot et al. 2004) after exposures to WTC dust.

PSD measurements demonstrated significant differences between FDNY-FF and TA-FF IS samples. We found a high load of relatively large particles (1–50 μm in diameter) with irregularly shaped structures in FDNY-FF IS samples that were completely different from the smaller, regular shaped particles found in TA-FF IS samples. Chemical and mineral analyses also demonstrated differences between FDNY-FF and TA-FF IS samples. TA-FF IS showed findings typical of soil contaminants from the Tel-Aviv area. In contrast, a heterogeneous mixture was found in FDNY-FF IS, consistent with exposures to aerosolized building debris and dust and smoke generated by the collapse and fires (Lioy et al. 2002). Mineral particles were seen in macrophages and epithelial cells. The presence of these particles in epithelial cells illustrates the high concentration of respirable particulate matter, overwhelming normal nasopharyngeal filtering, mucociliary clearance, and alveolar macrophage defense systems (Churg 1996). Although asbestos fibers were found in the BAL from the FDNY-FFs with eosinophilic pneumonitis after WTC exposure (Rom et al. 2002), we did not find asbestos fibers in our FDNY-FF IS samples.

Despite our finding significant correlations between inflammation and cumulative WTC exposure and significant differences in PSD between FDNY-FFs and TA-FFs, we could not detect a significant effect of cumulative WTC exposure on PSD. This may be related to other physical factors at the WTC site, such as differences in work location, minute ventilation (related to subject size, physical fitness, experience, and work tasks), specific work task–related exposures, and the use of respiratory protection (minimal during week one and variable thereafter) (CDC 2002b). It may also reflect potential limitations of scoring cumulative exposure only in terms of workdays, unweighted for the above differences and other variables such as environmental conditions. Additionally, selection bias (all FDNY-FF subjects volunteered for this study) may have influenced our current ability to detect the effect of cumulative exposure. However, the strengths of this study outweigh these limitations and include the following: *a*) all FDNY-FFs had significant WTC dust exposures because they were caught in the dust cloud during the collapse and then continued to work at the WTC site for days thereafter; *b*) IS provided an assessment of persistent inflammation and cumulative particle deposition because the enormity of the disaster prevented us from collecting IS until 10 months after the collapse; *c*) IS assessments were not biased toward acute, transient inflammation and particle deposition because at least 1 month had elapsed between collecting IS and the last workday at the WTC site; and *d*) controls were truly unexposed because they were recruited from outside this region—a necessity because by this time nearly every FDNY-FF and most other rescue workers and many Manhattan residents had reported some level of exposure.

We believe the differences between FDNY-FFs, TA-FFs, and controls demonstrate a unique exposure following the WTC collapse (Tables 3 and 4). This conclusion is supported by similarities between quantitative and qualitative analyses of IS sputum samples and dust samples collected from settled material one block east of the WTC. These dust samples demonstrate a complex mixture of coarse particles and fibers consisting of relatively larger particles (> 90% of dust particle mass was > 10 μm in diameter) (Lioy et al. 2002). Thus, the fact that IS samples from FDNY-FFs had higher amounts of particles > 2.0 μm in diameter than samples from TA-FFs was due to the nature and composition of the sources that dominated FDNY-FF exposure patterns. In 87% of FDNY-FFs, > 20% of the particles found in IS were > 2.0 μm in diameter compared with only 8% of those from TA-FFs. SEM also showed relatively larger and more irregularly shaped particles in FDNY-FFs compared with TA-FFs. Once inhaled, particles > 2.0 μm in diameter are most commonly deposited in the upper airways, causing significant irritation because of their alkaline, caustic nature (Lioy et al. 2002); this explains the increased incidence of upper airway symptoms (nasal congestion/drip, throat irritation, cough, and gastroesophageal reflux) described in highly and moderately exposed FDNY-FFs (Banauch et al. 2003; Feldman et al. 2004; Prezant et al. 2002).

Although helpful, measurements obtained from settled dust samples, which are often enriched with larger particles, are not entirely representative of the particle types or size distribution of aerosolized, potentially respirable dust during the height of exposure. In fact, the majority of particles in FDNY-FF IS samples were < 2.0 μm in diameter: 74% of FDNY-FFs had > 60% of PSD < 2.0 μm in diameter (Figure 2). Compared with settled WTC dust samples, FDNY-FF IS samples demonstrated a distinct leftward shift of the average curve of all PSD measurements toward smaller particles. Particles 2.0 μm in diameter are commonly deposited in the smaller airways. This may explain why post-WTC FDNY-FFs (Banauch et al. 2003; Prezant et al. 2002) have shown an increased incidence of bronchial hyperreactivity, reactive airways dysfunction syndrome, and asthma. Six months after the WTC collapse, methacholine challenge testing demonstrated that highly exposed FDNY rescue workers were 6.8 times more likely to have bronchial hyper-reactivity than moderately exposed and unexposed FDNY controls (Banauch et al. 2003).

Our findings support both the practical utility and scientific usefulness of IS as a non-invasive method for screening and follow-up monitoring of populations exposed to high concentrations of aerosolized particulates following a disaster—natural or man-made. IS is superior to BAL because it is noninvasive and collection can occur at nearly any field location. In contrast to traditional blood and urine biomonitoring, IS directly samples the lung, the specific target organ of interest following an inhalation exposure. For example, blood and urine samples were collected 1 month after the collapse in a different group of FDNY-FFs, and only a few of the 110 chemicals measured showed significant, yet small, differences when WTC-exposed FDNY-FFs were compared with nonexposed FDNY-FFs (Edelman et al. 2003). In contrast, IS directly assesses respired particulate matter and pulmonary inflammation, thereby serving an important complementary role to traditional biomonitoring techniques.

In conclusion, IS from FDNY-FFs caught in the WTC dust cloud during the morning of the collapse showed an influx of inflammatory cells, percentages of neutrophils and eosinophils that increased with exposure intensity, increased MMP-9 levels, a shift in PSD toward larger-size particles, and chemical/mineral analyses consistent with exposure to building debris, smoke, and dust generated by the attack on the WTC. If future population studies demonstrate that IS measures of inflammation or PSD correlate with health outcomes, then IS evaluations would be a valuable addition to medical screening/monitoring programs following inhalation exposures.

## Figures and Tables

**Table 1:** IS differential cell counts and MMP-9 levels.

	Macrophages (%)	Neutrophils (%)	Lymphocytes (%)	Eosinophils (%)	MMP-9 (ng/mL)
FDNY-FF (*n* = 39)	34.3 ± 15.2	50.7 ± 17	12 ± 6.8	2.8 ± 4	2.2 ± 2.6
TA-FF (*n* = 12)	35 ± 18.9	44.1 ± 22.9	15.8 ± 9.8	5.4 ± 8.9	1.2 ± 0.99*
Control (*n* = 8)	64.8 ± 7.9**	29.1 ± 8.9**	5.7 ± 2.2**	0.2 ± 0.4**	0.3 ± 0.09**

Differential counts are expressed as a percentage of 200 cells as described in “Methods.”

* *p* = 0.057 between levels in FDNY-FFs versus TA-FFs.

** *p* < 0.05 between cells in FDNY-FFs and TA-FFs versus controls.

**Table 2:** Differential counts, MMP-9 levels, and particle size distribution in FDNY-FFs analyzed according to cumulative exposure.

	Cumulative workdays at the WTC		
	< 10 days	≥ 10 days	*p*-Value
Duty (years)	15.6 ± 8.3	17.6 ± 8.6	0.78
Macrophages (%)	31.1 ± 13.7	38.4 ± 16.5	0.18
Neutrophils (%)	44.2 ± 16.5	55.7 ± 15.2	0.05
Lymphocytes (%)	11.6 ± 7.0	12.9 ± 6.8	0.59
Eosinophils (%)	1.5 ± 1.9	4.4 ± 5.2	0.04
Particles > 2 μm (%)	32.3 ± 13.7	38.6 ± 17	0.48
Particles > 5 μm (%)	7.8 ± 3.3	9.3 ± 7.1	0.28
MMP-9 (ng/mL)	1.73 ± 0.98	2.7 ± 4.0	0.36

Differential cell counts are expressed as a percentage of 200 cells as described in “Methods.”

**Table 3:** Particle analysis in IS of FDNY-FFs and TA-FFs.

Measurement	FDNY-FF	TA-FF	*p*-Value
Percent of particles > 2 μm*^a^*	34.01 ± 15.5	10.4 ± 5.8	*p* = 0.0001
Percent of particles > 5 μm*^a^*	8. 1 ± 15.3	1.6 ± 1.25	*p* = 0.0001
Percent of samples with > 20% particles > 2 μm*^b^*	88.6% (31/35)	8.3% (1/12)	*p* = 0.0001
Percent of samples with > 5% particles > 5 μm*^b^*	85.7% (30/35)	0% (0/12)	*p* = 0.0001

a Percentage of total particles present in the sample.

b Percentage of total IS samples measured by a Cis-100 analyzer in each group (*n* = 35 FDNY-FFs; *n* = 12 TA-FFs) as described in “Methods.”

**Table 4:** Mineralogic analysis of IS particles from FDNY-FFs and TA-FFs.

Subject no.	Particle size (μm)	Frequent elements	Type of particles
FDNY-FF 16	1.5–50	Si, SiCa, SiFe	Silica
		TiFe, Ti	Titanium oxide
		FeNi, FeCr	Stainless steel
		Ca	Calcite
FDNY-FF 3	1.5–50	Zn	Zinc oxide
		Ca	Calcite
FDNY-FF 26	1.0–10	Si, SiCa	Silica
		SiFeNi, SiFe	Ferrous alloys
FDNY-FF 35	1.0–50	Ca	Calcite
		MgAlSiCa, AlSiCa, AlSi	Clays
		Zn	Zinc oxide
		AgSnCuHg	Nonferrous alloys
TA-FF 10	0.8–7.0	FeTi	Stainless steel
		Si	Silica
		AlSiCaFe, AlSiFe	Clays
		AlSiFeTi, AlSi	Clays
		MgSiAlFe	Clays
TA-FF 12	3–10	Fe	Ferric oxide
		FeCa, FeCr	Stainless steel

Analysis was performed as described in “Methods.”

## References

[b1-ehp0112-001564] Aharonson EF, Karasikov N, Roitberg M, Shamir J (1986). GALAI-CIS-1—a novel approach to aerosol partical size analysis. J Aerosol Sci.

[b2-ehp0112-001564] Atkinson JJ, Senior RM (2003). Matrix metalloproteinase-9 in lung remodeling. Am J Respir Cell Mol Biol.

[b3-ehp0112-001564] Azadniv M, Torres A, Boscia J, Speers DM, Frasier LM, Utell MJ (2001). Neutrophils in lung inflammation: which reactive oxygen species are being measured?. Inhal Toxicol.

[b4-ehp0112-001564] Banauch GI, Alleyne D, Sanchez R, Olender K, Cohen HW, Weiden M (2003). Persistent hyperreactivity and reactive airway dysfunction in firefighters at the World Trade Center. Am J Respir Crit Care Med.

[b5-ehp0112-001564] Bergstrom CE, Eklund A, Skold M, Tornling G (1997). Bronchoalveolar lavage findings in firefighters. Am J Ind Med.

[b6-ehp0112-001564] Burgess JL, Nanson CJ, Bolstad-Johnson DM, Hysong TA, Sherrill DL, Quan SF (2001). Adverse respiratory effects following overhaul in firefighters. J Occup Environ Med.

[b7-ehp0112-001564] Cataldo DD, Bettiol J, Noel A, Bartsch P, Foidart JM, Louis R (2002). Matrix metalloproteinase-9, but not tissue inhibitor of matrix metalloproteinase-1, increases in the sputum from allergic asthmatic patients after allergen challenge. Chest.

[b8-ehp0112-001564] CDC (Centers for Disease Control and Prevention) (2002a). Occupational exposures to air contaminants at the World Trade Center disaster site - New York, September - October 2001. MMWR.

[b9-ehp0112-001564] CDC (Centers for Disease Control and Prevention) (2002b). Use of respiratory protection among responders at the World Trade Center Site - New York City, September 2001. MMWR.

[b10-ehp0112-001564] CDC (Centers for Disease Control and Prevention) (2002c). Self-reported increase in asthma severity after the September 11 attacks on the World Trade Center - Manhattan, New York 2001. MMWR.

[b11-ehp0112-001564] Churg A (1996). The uptake of mineral particles by pulmonary epithelial cells. Am J Respir Crit Care Med.

[b12-ehp0112-001564] Cohen C, Fireman E, Ganor E, Man A, Ribak J, Lerman Y (1999). Accelerated silicosis with mixed-dust pneumoconiosis in a hard-metal grinder. J Occup Environ Med.

[b13-ehp0112-001564] Davison AG, Haslam PL, Corrin B, Coutts II, Dewar A, Riding WD (1983). Interstitial lung diseases and asthma in hard metal workers: bronchoalveolar lavage, ultrastructure, and analytical findings and results of bronchial provocation tests. Thorax.

[b14-ehp0112-001564] Dodson FR, Garcia GN, O’Sullivan M, Corn C, Levin JL, Griffith DE (1991). The usefulness of bronchoalveolar lavage in identifying past occupational exposure to asbestos: a light and electron microscopy study. Am J Ind Med.

[b15-ehp0112-001564] Edelman P, Osterloh J, Pirkle J, Caudill SP, Grainger J, Jones R (2003). Biomonitoring of chemical exposure among New York City firefighters responding to the World Trade Center fire and collapse. Environ Health Perspect.

[b16-ehp0112-001564] Feldman DM, Baron SL, Bernard BP, Lushniak BD, Banauch G, Arcentales N (2004). Symptoms, respirator use, and pulmonary function changes among New York City firefighters responding to the World Trade Center disaster. Chest.

[b17-ehp0112-001564] Fireman E, Goshen M, Ganor E, Lerman Y (2004a). Induced sputum as an additional tool in the identification of metal-induced sarcoid-like reaction. Sarcoidosis Vasc Diffuse Lung Dis.

[b18-ehp0112-001564] Fireman E, Greif J, Schwarz Y, Man A, Ganor E, Ribak Y (1999a). Assessment of hazardous dust exposure by BAL and induced sputum. Chest.

[b19-ehp0112-001564] Fireman E, Moscovich A, Lerman Y (2004b). High metalloproteinase 9 levels in induced sputum unexpectedly associated with smoking among workers exposed to hazardous dust [Abstract]. Am J Respir Crit Care Med.

[b20-ehp0112-001564] Fireman E, Topilsky I, Greif J, Lerman Y, Schwarz Y, Man A (1999b). Induced sputum compared to bronchoalveolar lavage for evaluating patients with sarcoidosis and non-granulomatous interstitial lung disease. Respir Med.

[b21-ehp0112-001564] Landrigan PJ, Lioy PJ, Thurston G, Berkowitz G, Chen LC, Chillrud SN (2004). Health and environmental consequences of the World Trade Center disaster. Environ Health Perspect.

[b22-ehp0112-001564] Lerman Y, Schwarz Y, Kaufman G, Ganor E, Fireman E (2003a). Case series: use of induced sputum in the evaluation of occupational lung diseases. Arch Environ Health.

[b23-ehp0112-001564] Lerman Y, Segal B, Rochvarger M, Winberg D, Kivity O, Fireman E (2003b). Particles size distribution in induced sputum and pulmonary function among foundry workers. Arch Environmental Health.

[b24-ehp0112-001564] Lemiere C, Chaboilez S, Malo JL, Cartier A (2001). Changes in sputum cell counts after exposure to occupational agents: what do they mean?. J Allergy Clin Immunol.

[b25-ehp0112-001564] Li Q, Park PW, Wilson CL, Parks WC (2002). Matrilysin shedding of syndecan-1 regulates chemokine mobilization and transepithelial efflux of neutrophils in acute lung injury. Cell.

[b26-ehp0112-001564] Lioy PJ, Weisel CP, Millette JR, Eisenreich S, Vallero D, Offenberg J (2002). Characterization of the dust/smoke aerosol that settled east of the World Trade Center (WTC) in lower Manhattan after the collapse of the WTC 11 September 2001. Environ Health Perspect.

[b27-ehp0112-001564] Maestrelli P, Calcagni PG, Saetta M, DiStefano A, Hosselet JJ, Santonastaso A (1994). Sputum eosinophilia after asthmatic responses induced by isocyanates in sensitized subjects. Clin Exp Allergy.

[b28-ehp0112-001564] Marek W, Kotschy-Lang N, Muti A, Kohler CH, Nielsen L, Topalidis TH (2001). Can semi-automated image cytometry on induced sputum become a screening tool for lung cancer? Evaluation of quantitative semi-automated sputum cytometry on radon- and uranium-exposed workers. Eur Respir J.

[b29-ehp0112-001564] Montano M, Beccerril C, Ruiz V, Ramos C, Sansores RH, Gonzalez-Avila G (2004). Matrix metalloproteinases activity in COPD associated with wood smoke. Chest.

[b30-ehp0112-001564] Murphy G, Docherty AJP (1992). The matrix metalloproteinases and their inhibitors. Am J Respir Cell Mol Biol.

[b31-ehp0112-001564] Paris C, Galateau-Salle F, Creveuil C, Morello R, Raffaelli C, Gillon J (2002). Asbestos bodies in the sputum of asbestos workers: correlation with occupational exposure. Eur Respir J.

[b32-ehp0112-001564] Pin I, Gibson PG, Kolendowich R, Girgis Gabardo A, Denburg JA, Hargreave FE (1992). Use of induced sputum cell counts to investigate airway inflammation in asthma. Thorax.

[b33-ehp0112-001564] Popov T, Gottschalk R, Kolendowich R, Dolovich J, Powers P, Hargreave FE (1994). The evaluation of a cell dispersion method of sputum examination. Clin Exp Allergy.

[b34-ehp0112-001564] Prezant DJ, Weiden M, Banauch GI, McGuinness G, Rom WN, Aldrich TK (2002). Cough and bronchial responsiveness in firefighters at the World Trade Center site. N Engl J Med.

[b35-ehp0112-001564] Quirce S, Baeza ML, Tornero P, Blasco A, Barranco R, Sastre J (2001). Occupational asthma caused by exposure to cyanoacrylate. Allergy.

[b36-ehp0112-001564] Rom WN, Weiden M, Garcia R, Yie TA, Vathesatogkit P, Tse DB (2002). Acute eosinophilic pneumonia in a New York City firefighter exposed to World Trade Center dust. Am J Respir Crit Care Med.

[b37-ehp0112-001564] Safirstein BH, Klukowic A, Miller R, Teirstein A (2003). Granulomatous pneumonitis following exposure to the World Trade Center collapse. Chest.

[b38-ehp0112-001564] Saltzman SH, Moosavy FM, Misskoff JA, Friedmann P, Fried G, Rosen MJ (2004). Early respiratory abnormalities in emergency services police officers at the World Trade Center site. J Occup Environ Med.

[b39-ehp0112-001564] Skloot G, Goldman M, Fischler D, Goldman C, Shecter C, Levin S (2004). Respiratory symptoms and physiologic assessment of ironworkers at the World Trade Center disaster site. Chest.

[b40-ehp0112-001564] Szema AM, Khedar M, Maloney PF, Tackach PA, Nickels MS, Patel H (2004). Clinical deterioration in pediatric asthmatic patients after September 11, 2002. J Allergy Clin Immunol.

[b41-ehp0112-001564] Woodruff PG, Khashayar R, Lazarus SC, Janson S, Avila P, Boushey HA (2001). Relationship between airway inflammation, hyperresponsiveness and obstruction in asthma. J Allergy Clin Immunol.

